# Expression of HSPA14 in patients with acute HIV-1 infection and its effect on HIV-1 replication

**DOI:** 10.3389/fimmu.2023.1123600

**Published:** 2023-02-09

**Authors:** Mingyuan Bi, Wen Kang, Yongtao Sun

**Affiliations:** Department of Infectious Diseases, Tangdu Hospital, Air Force Medical University, Xi’an, China

**Keywords:** HIV, replication, HSPA14, HspBP1, HIV RNA

## Abstract

**Introduction:**

Heat shock protein (HSPs) are important intracellular factors, which are often involved in the regulation of viral replication including HIV-1 in infected individuals as molecular chaperone proteins. Heat shock proteins 70 (HSP70/HSPA) family play important roles in HIV replication, but this family contain many subtypes, and it is unclear how these subtypes participate in and affect HIV replication.

**Methods:**

To detect the interaction between HSPA14 and HspBP1 by CO-IP. Simulating HIV infection status *in vitro* to detect the change of intracellular HSPA14 expression after HIV infection in different cells. Constructing HSPA14 overexpression or knockdown cells to detect intracellular HIV replication levels after *in vitro* infection. Detecting the difference of HSPA expression levels in CD4+ T cells of untreated acute HIV-infected patients with different viral load.

**Results:**

In this study, we found that HIV infection can lead to changes in the transcriptional level of many HSPA subtypes, among which HSPA14 interacts with HIV transcriptional inhibitor HspBP1. The expression of HSPA14 in Jurkat and primary CD4+T cells infected with HIV were inhibited, overexpression of HSPA14 inhibited HIV replication, while knocking down HSPA14 promoted HIV replication. We also found that the expression level of HSPA14 is higher in peripheral blood CD4+T cells of untreated acute HIV infection patients with low viral load.

**Conclusion:**

HSPA14 is a potential HIV replication inhibitor and may restrict HIV replication by regulating the transcriptional inhibitor HspBP1. Further studies are needed to determine the specific mechanism by which HSPA14 regulates viral replication

## 1 Introduction

After acute HIV infection, there is a major difference in the distribution of HIV RNA and HIV DNA levels in patients, and the high level of virus replication can lead to continuous immune activation, inflammation and progressive damage to the immune function. HIV RNA is a common indicator of the level of virus replication in patients (1). Clinical statistics show that the HIV RNA level in plasma of untreated acute infection is usually 0.25–95.5 × 10^5^ copies/ml (2, 3). The difference in virological level among acute HIV infection patients without treatment is mainly due to two aspects: the characteristics of the virus subtypes and the autoimmune characteristics of the infected individuals. The main virus subtypes in China are the AE recombinant type and BC recombinant type, thus the influence of virus subtypes on HIV replication levels is limited (4), and the level of HIV replication in patients may be mainly regulated by individual host factors (5, 6).

The heat shock protein (HSP) family is a kind of chaperone protein family expressed in many cells, and its role in HIV replication has been widely studied (7–9). Among them, the HSP70 (also called HSPA) subtribe plays an important role in the process of HIV replication. HSPA subtribe exists in various human cells with more than 14 different isoforms, including heat-inducible isoforms (e.g., HSPA1A, HSPA1B, HSPA6, HSPA7, etc.) and non-heat-inducible isoforms (e.g., HSPA13). The subtype-specific roles of HSPAs have been extensively studied. HSPA5 was found to be involved in the folding process of the viral Env protein in the endoplasmic reticulum, while other HSPA isoforms have different interactions with viral proteins such as Tat, Gag, and Vpr (10, 11). Kammula et al. demonstrated the interaction of HSPA9 with the viral Nef protein through a yeast two-hybrid screen, suggesting that HSPA9 could be able to participate in the regulation of HIV replication (12). HSPA14 was first identified in dendritic cells, and it is also widely found in a variety of immune cells such as T lymphocytes and monocytes, playing a role in enhancing Th1-polarizing activity. However, whether HSPA14 can interfere with HIV replication remains unclear. In the current study, we investigated the effect of HSPA14 in regulation of HIV replication. We now report, for the first time, that HSPA14 is an important inhibitory factor for HIV infection in T cells.

## 2 Materials and methods

### 2.1 Study subjects

This study was approved by the Medical Ethics Committee of Tangdu Hospital (second affiliated Hospital) of Air Force military Medical University. Untreated acute HIV infection patients came from Department of Infectious Diseases of Tangdu Hospital. The normal seronegative donors came from Department of Blood Transfusion of Tangdu Hospital. This study collects at least 70 cases of untreated acute HIV infection patients and 20 healthy controls, aged from 18 to 60 years old, regardless of gender and excluded trauma, tumor, autoimmune disease or other pathogen infection. Untreated acute HIV infection patients are divided into high viral load group (HVL) and low viral load group (LVL) according to whether HIV RNA level is greater than 1 × 10^5^ IU/ml, and there’s no significant difference in baseline characteristics between two groups (**Table 1**).

**Table 1:** Comparison of Baseline Characteristics Between the HVL and LVL Groups.

Baseline characteristics	HVL	LVL	*t*/χ^2^ value	*P* value
Number	34	36	/	/
Gender (male/female)	30/4	32/4	0.572	0.682
Age (years)	30.32 ± 7.13	29.12 ± 7.84	0.312	0.781
Infection time (days)	24.54 ± 3.12	23.19 ± 2.54	1.183	0.242
Account of CD4^+^T cells (cells/μl)	356.31 ± 205.44	395.23 ± 240.37	0.686	0.525
Account of CD8^+^T cells (cells/μl)	1186.76 ± 615.54	1223.15 ± 488.27	0.198	0.843
CD4^+^/CD8^+^	0.31 ± 0.14	0.31 ± 0.16	0.149	0.881

### 2.2 Pseudovirus preparation

HIV pseudoviral particles were obtained by ultracentrifugation after co-transfection of plasmid pNL-4-3murmurRmure- (From our laboratory) and plasmid pCMV-VSVG (Genechem, China) in HEK293T cells by lipofection (Lipo3000, Invitrogen™, USA), and cultured in a 5% CO_2_, 37°C cell incubator for 48-72 hours. Collect the cell culture supernatant, centrifuge at 20000×g for 10 minutes at room temperature, and then filter the supernatant with a 0.2 µm filter to obtain the HIV-1 pseudovirus solution. TZM-bl cells were tiled in a 96-well plate and prepared to detect the TCID50 of HIV-1 pseudovirus. Dilute the HIV-1 pseudovirus solution according to a 10-fold gradient, add 100 µL of the solution dropwise to a row of microwells, and continue culturing the 96-well plate after adding HIV-1 pseudovirus solutions of different dilution levels in a 5% CO_2_, 37°C cell culture incubator for 24 hours. Observe and record the cell state of each microwell in the 96-well plate, and calculate the TCID50 of HIV-1 pseudovirus according to the Reed&Muench method (**Table 2**).

**Table 2:** Calculate the TCID50 of HIV-1 pseudovirus by Reed&Muench method.

Index	Dilution series					
	10^-1^	10^-2^	10^-3^	10^-4^	10^-5^	10^-6^
Total wells	8	8	8	8	8	8
Positive wells	8	7	4	4	3	1
Negative wells	0	1	4	4	5	7
Cum pos	27	19	12	8	4	1
Cum neg	0	1	5	9	14	21
Infected (%)	100.00	95.00	70.59	47.06	22.22	4.55
Prop dist	0.00	1.84	0.87	-0.12	-1.57	-10.00
TCID50	1.33×10^-4^					
TCID50/mL	7.50×10^4^					

### 2.3 Pseudovirus HIV-1 infection of cell lines

CCRF-CEM cell line (Procell, China) and Jurkat cell line (Procell, China) were cultured in RPMI-1640 complete medium [500mlRPMI-1640+50ml fetal bovine serum (FBS) + penicillin streptomycin] at 37°C and 5%CO_2_. HEK-293T cell line (Procell, China) and TZM-bl cell line (From our laboratory) were cultured in DMEM complete medium [500mlDMEM+50ml fetal bovine serum (FBS) + penicillin streptomycin] at 37°C and 5%CO_2_. Peripheral blood mononuclear cells (PBMC) were isolated by Ficoll density gradient centrifugation from blood samples of patients. Commercial CD4+T lymphocyte separation kit (STEMCELL, Canada) was used to isolate CD4+T lymphocytes by negative selection. CD4+T lymphocytes were short-term cultured in RPMI-1640 complete medium [500mlRPMI-1640+50ml fetal bovine serum (FBS) + penicillin streptomycin] at 37°C and 5%CO_2_. When used in the experiment of infected cells, different cells were inoculated on the culture plate according to the following number of cells: 10^4^/well-96-well plate, 5 × 10^4^/well-24-well plate, 2.5 × 10^5^/well-6-well plate. Using the measured virus titer TCID50/ml, combined with the formula PFU=0.7 × TCID50/ml, MOI=PFU/cell number, when the cell confluence degree reached 60%-70%, the original culture medium was discarded and washed with DPBS for 2 times, and the required amount of virus was added, and the serum-free medium was added. After being cultured in 37°C, CO_2_ incubator for 2 hours, the complete medium was changed, and then the next experiment was carried out according to different research purposes.

### 2.4 Overexpression and knockdown of HSPA14

The overexpression plasmids of HSPA14 (GV657 vector) have been provided by *Genechem, China*. The recombinant product of HSPA14 was transformed into competent bacteria, and the plasmid was obtained by expanding culture and extraction of the correct clone solution. The tool vector plasmid carrying the HSPA14 gene and the virus packaging helper plasmid were co-transfected into HEK293T cells by lipofection (Lipo3000, Invitrogen™, USA). The virus was harvested 48-72 hours after transfection. The lentivirus preservation solution with high titer was obtained by PEG8000 precipitation, concentration and purification, and the lentivirus titer was determined. The obtained lentivirus infection requires cells that overexpress HSPA14, and cells stably expressing HSPA14 can be obtained for follow-up experiments.

According to the nucleotide sequences of HSPA14, the short hairpin RNA (shRNA, GV493 vector) was synthesized by chemical synthesis method from *Genechem, China*. The target cells in logarithmic growth phase were inoculated with a density of about 50%, and cultured overnight in an incubator with a volume fraction of 5% CO_2_ at 37°C. The transfection solution was added to cells and cultured in an incubator with a volume fraction of 5% CO_2_ at 37°C. After 4 hours, the culture medium was discarded and replaced with 10% fetal bovine serum and DMEM culture medium without antibiotics. 48 hours after transfection, the cells were digested with trypsin by hole, and RNA was extracted to detect the interference efficiency after washing with PBS.

For both overexpression and interference with HSPA14, cell lines transfected with vector plasmids (GV657 vector or GV493 vector) were set up as Controls, and cell lines without transfection were used as Blank Controls.

### 2.5 Immunoblotting and immunoprecipitation assays

Total cellular proteins were extracted by RIPA lysis buffer (Thermo Scientific™, USA) with protease inhibitor (Thermo Scientific™, USA). The protein concentration was determined by the Rapid Gold BCA Protein Assay Kit (Thermo Scientific™, USA), and the same amount of protein was detected on a NuPAGE 10% Mini Protein Gels (Invitrogen™, USA) by vertical plate electrophoresis tank (Bio-Rad, USA). Then the proteins were transferred from gels to PVDF transfer membranes (Thermo Scientific™, USA) by Trans-Blot SD Semi-Dry transfer tank (Bio-Rad, USA), blocked with 5% skim dried milk or BSA (Thermo Scientific™, USA), and detected with the corresponding antibody (**Table 3**). Imprinting was performed with the ECL Chemiluminescent Substrate Reagent Kit (Invitrogen™, USA). For coimmunoprecipitation analysis, the total proteins of the corresponding cells were incubated with HSPA14 or HspBP1 antibodies, and the antigen-antibody complex was pulled down by Classic Magnetic IP/Co-IP Kit (Thermo Scientific™, USA) and then identified by the immunoblotting method described above.

**Table 3:** List of the antibodies used for immunoblotting and immunoprecipitation assays.

Antibodies name	Optimal dilutions (Concentration)	Vender	Catalog number
HSPA2	WB 1:5000	Invitrogen™, USA	PA5-117993
HSPA5(GRP78)	WB 1:2000	Invitrogen™, USA	PA5-24963
HSPA6	WB 1:2000	Invitrogen™, USA	PA5-103416
HSPA7	WB 1:2000	Invitrogen™, USA	PA5-35359
HSPA8(HSC70)	WB 1:2000	Invitrogen™, USA	PA5-116380
HSPA9	WB 1:1500	Invitrogen™, USA	PA5-86148
HSPA13(STCH)	WB 1:2000	Invitrogen™, USA	PA5-100565
HSPA14	WB 1:1500 IP 3 μL/mg of lysate	Invitrogen™, USA	PA5-117992
HspBP1	WB 1:1500 IP 1:20	Invitrogen™, USA	PA5-35155
p24	WB 1:1500	Invitrogen™, USA	PA1-7217
GAPDH	WB 1:1500	Invitrogen™, USA	A300-640A-T
β-actin	WB 1:5000	Invitrogen™, USA	PA1-16889

### 2.6 Enzyme-linked immunosorbent assay

The level of HIV p24 in the supernatant of different cell lines infected by HIV pseudovirus was measured by HIV-1 p24 SimpleStep ELISA Kit (Abcam, UK) as follows. (1) Prepare standards and cell culture supernatants according to the protocol. (2) Prepare all regents and add 50µL of all samples and standards to plate wells. (3) Add 50µL of the Antibody Cocktail to each well and incubate for 1 hour on a plate shaker set to 400 rpm (4) Thoroughly wash each well with 1×Wash Buffer PT and remove excess liquid (5) Add 100µL of TMB Development Solution to each well and incubate for 10 minutes in the dark on a plate shaker set to 400 rpm (6) Add 100 µL of Stop Solution to each well and shake plate on a plate shaker for 1 minute to mix. (7) Record the OD of each well at 450 nm and calculate the concentration of HIV p24 in each sample by standard curve.

### 2.7 Total cQuantitative real-time PCR

An RNA Easy Fast animal tissue/cell total RNA extraction kit (TIANGEN, China) was used to extract total RNA from cells. cDNA was prepared by a RevertAid First Strand cDNA Synthesis Kit (Thermo Scientific™, USA). The expression of HSPAs and p24 was detected by real-time quantitative polymerase chain reaction, and the corresponding forward and reverse primer sequences shown in **Table 4**. The qRT-PCR procedure is as follows: (1) preincubation: 95°C, ramp 4.4°C/s, 600s; (2) Three-step amplification for 45 cyclers: 95°C, ramp 4.4°C/s, 10s; 60°C, ramp 2.2°C/s, 10s; 72°C, acquisition with ramp 4.4°C/s, 10s; (3) Melting: 95°C, ramp 4.4°C/s, 10s; 65°C, ramp 2.2°C/s, 60s; 97°C, ramp 0.1°C/s, 1s. Finally, the CT value of the corresponding reaction well was obtained, and the 2-Δ(ΔCT) value was calculated by the following formula, which yields the degree of change in the relevant HSPA mRNA level of the corresponding sample.

**Table 4:** List of the primers used for qRT-PCR analysis.

Primer Name	Primer Sequences	Number of bases	Purification Type
HSPA2 RT FP	GACAACCAGAGCAGCGTACT	20	PAGE
HSPA2 RT RP	CATCCGGTCAATGTCGTCCT	20	PAGE
HSPA5 RT FP	CACAGTGGTGCCTACCAAGA	20	PAGE
HSPA5 RT RP	TGATTGTCTTTTGTCAGGGGT	21	PAGE
HSPA6 RT FP	CGTGCCCGCCTATTTCAATG	20	PAGE
HSPA6 RT RP	AAAAATGAGCACGTTGCGCT	20	PAGE
HSPA7 RT FP	GCCTGGTGTGCTTATTCAGG	20	PAGE
HSPA7 RT RP	ACGGCCCTTGTCATTAGTGA	20	PAGE
HSPA8 RT FP	TTACCCGTGCCCGATTTGAA	20	PAGE
HSPA8 RT RP	CCTGGCATGCCTGTGAGTT	19	PAGE
HSPA9 RT FP	GTGGCCTTTACAGCAGATGG	20	PAGE
HSPA9 RT RP	GCTCCAATCTGACTCGGAGA	20	PAGE
HSPA13 RT FP	CCATCACAGTGTCCCCAGAA	20	PAGE
HSPA13 RT RP	CTGCTGCTGTGGGTTCATTT	20	PAGE
HSPA14 RT FP	TTGCAAATGATGCCGGTGAC	20	PAGE
HSPA14 RT RP	CCTCTGAAAAGGCTGTGGGA	20	PAGE
p24 RT FP	GACAACCAGAGCAGCGTACT	20	PAGE
p24 RT RP	CATCCGGTCAATGTCGTCCT	20	PAGE
β-actin-RT-FP	GCTACGTCGCCCTGGACTTC	20	PAGE
β-actin-RT-RP	GTCATAGTCCGCCTAGAAGC	20	PAGE

Fold Change = 2^-Δ(ΔCT)^

where ΔCT = CT(target) – CT(β-actin)

and Δ(ΔCT) = ΔCT(treated) – ΔCT(control)

### 2.8 Statistical analysis

SPSS 23.0 was used for statistical analysis, GraphPad Prism 8.0 was used for drawing graphs. All immunoblotting experiments and qRT-PCR experiments were carried out in 3 biological replicates x̄ ± s was used to depict the measurement data, and the measurement data between different groups were compared by *Student’s t test*. The enumeration data are described by n (%), and the *chi-square test* was used to compare enumeration data between different groups. When P < 0.05, the difference between groups was considered statistically significant.

## 3 Result

### 3.1 Interaction between HspBP1 and HSPA14

**Figure 1:**
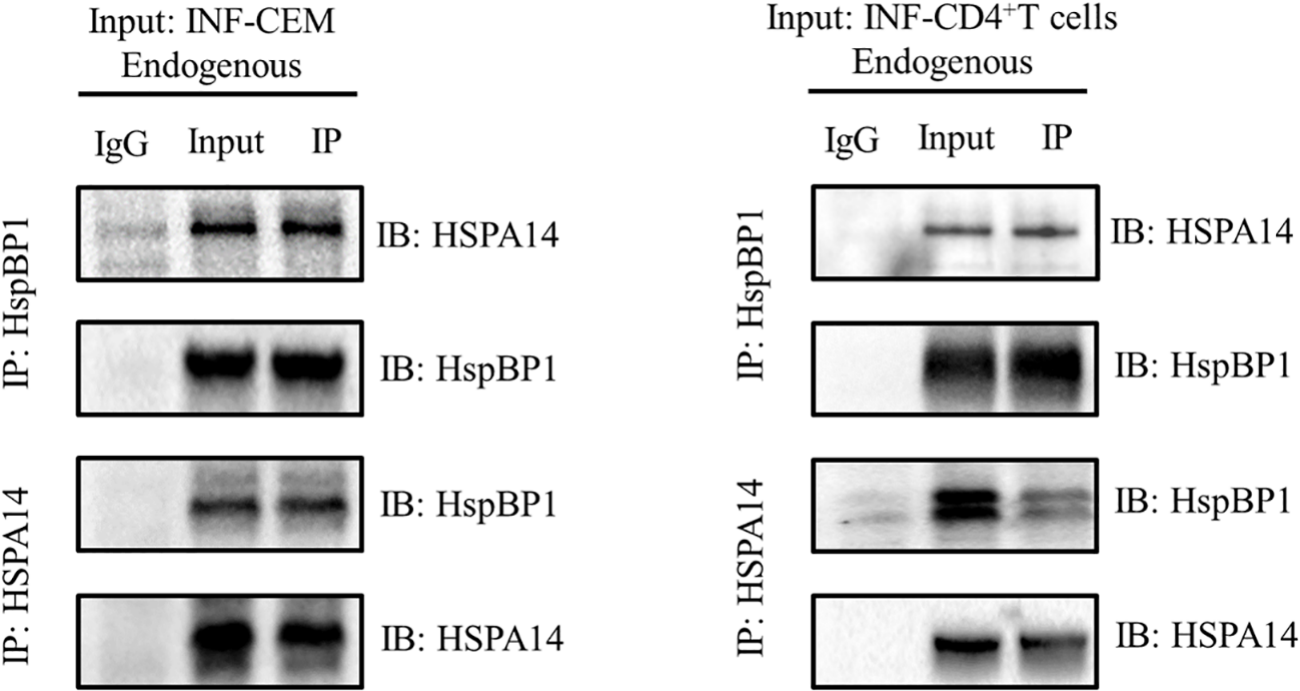
Co-IP to detect the interaction between HspBP1 and HSPA14. Co-immunoprecipitation showing interaction of HspBP1 with HSPA14 at endogenous level in infected CEM cells and CD4+T cells of acute HIV infected patients.

HspBP1 is known to play an important role in interfering HIV transcription and interact with some subtypes of HSP70s. Taking these observations into consideration, we investigated whether HSPA14 interacts with HspBP1 in HIV infected cells. To accomplish this, we performed co-immunoprecipitation assays with lysate from CEM cells infected with HIV pseudovirus and CD4+T cells isolated from acute HIV infected patients PBMC. We observed that HSPA14 was pulled down with HspBP1 antibodies. Likewise, HspBP1 was also pulled down with HSPA14 antibody (**Figure 1**), suggesting that HSPA14 can interact with HspBP1.

### 3.2 HSPA14 is down-modulated during HIV-1 infection

**Figure 2:**
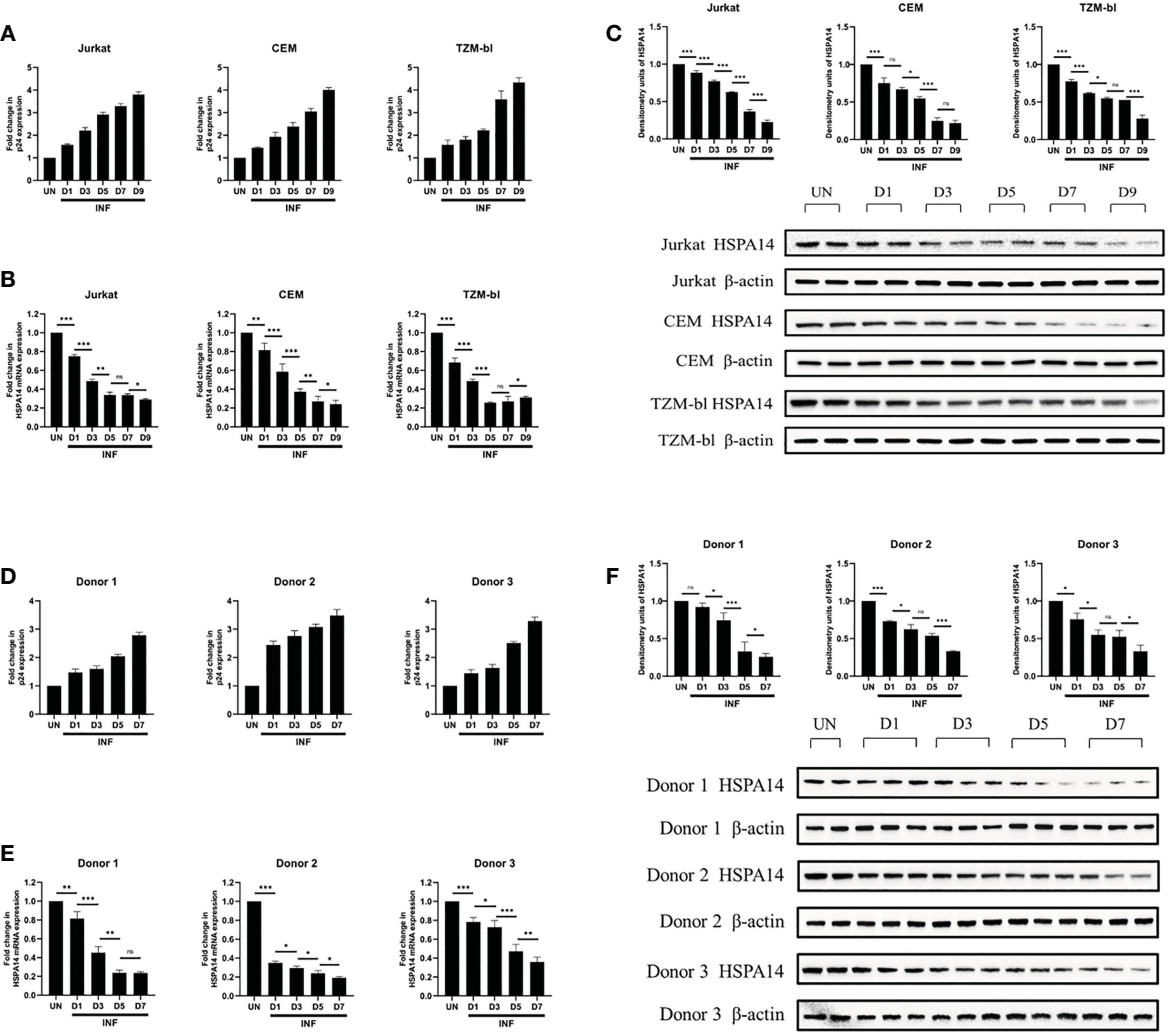
HSPA14 is down modulated during HIV-1 infection. **(A, D)** The expression of viral capsid protein p24 mRNA was measured to monitor the progression of infection. **(B)** mRNA expression profiling of HSPA14 during HIV infection. Different cells were infected with HIV pseudovirus and were harvested on day 1 (D1), day 3 (D3), day 5 (D5), day 7 (D7) and day 9 (D9), post infection for quantitative real time PCR analysis. **(C)** Representative western blot and densitometry analysis showing expression profile of HSPA14 in HIV pseudovirus infected cells at different time points. **(E)** mRNA expression profiling of HSPA14 during HIV infection. Different cells were infected with HIV pseudovirus and were harvested on day 1 (D1), day 3 (D3), day 5 (D5) and day 7 (D7), post infection for quantitative real time PCR analysis. **(F)** Representative western blot and densitometry analysis showing expression profile of HSPA14 in HIV pseudovirus infected CD4+T cells at different time points. CD4+T cells were isolated from PBMC of three healthy donors and were infected with HIV pseudovirus. The error bars are presented as the mean ± SD values and significance is defined as *P ≤ 0.05, **P ≤ 0.01 and ***P ≤ 0.001. INF: Infected with HIV-1. UN, Uninfected.

We next examined HSPA14 expression levels during HIV-1 infection. Considering the interference of HspBP1 to HIV replication and evidence from **Figure 1** that HSPA14 interacts with HspBP1, we were curious to know that what happens to HSPA14 levels during infection. Kinetic expression profile studies for HSPA14 were performed following HIV-1 infection in Jurkat cells, CEM cells and TZM-bl cells. Different cells were infected with HIV pseudovirus and expression of HSPA14 was analyzed at mRNA and protein level at various time points. HIV-1 infection resulted in down-modulation of HSPA14 mRNA and protein expression (**Figures 2A–C**). This finding was further confirmed by analyzing the expression of HSPA14 in HIV infected human CD4+T cells isolated from the PBMC of three normal seronegative donors (**Figures 2D–F**). These results clearly indicate that HSPA14 mRNA and protein levels are significantly down-modulated following HIV infection.

### 3.3 HSPA14 interferes with HIV-1 replication

**Figure 3:**
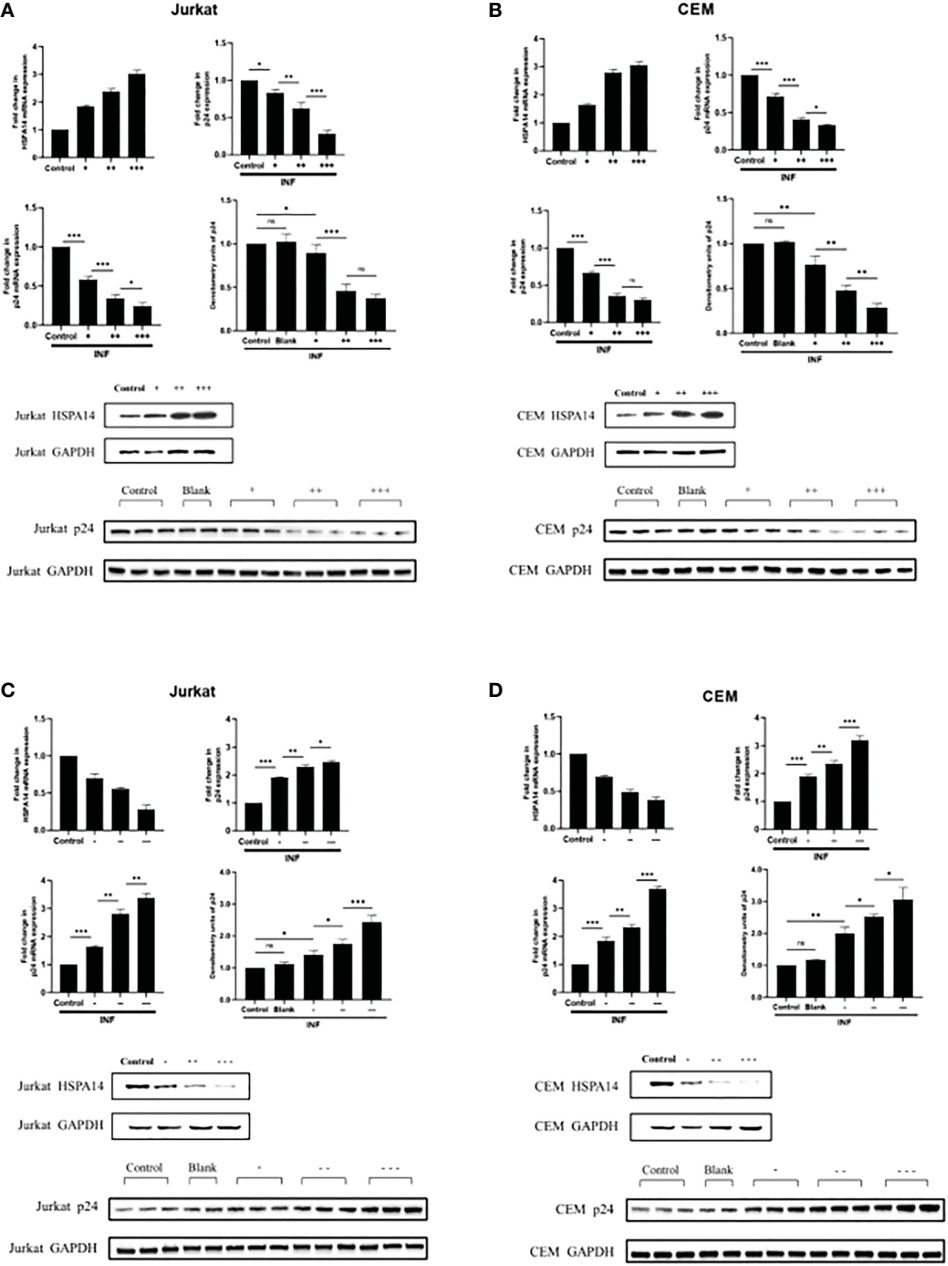
HSPA14 can interfere the HIV-1 replication **(A)** Jurkat cells were transfected with indicated plasmids. 24 hours post-transfection, cells were infected with HIV pseudovirus. 24 hours post-infection, cells were harvested to isolate total RNA and protein, and culture supernatants were collected. Upper left panel: qRT-PCR analysis showing different dose of HSP14 over-expression in Jurkat cells. Upper right panel: Culture supernatants were examined for amount of virus produced using p24 antigen capture ELISA. Middle left panel: qRT-PCR analysis revealing change of p24 mRNA levels. Middle right panel and lower panel: representative western blot and densitometry analysis revealing change of p24 protein levels. **(B)** CEM cells were transfected with indicated plasmids as Jurkat cells. Upper left panel: qRT-PCR analysis showing different dose of HSP14 over-expression in CEM cells. Upper right panel: Culture supernatants were examined for amount of virus produced using p24 antigen capture ELISA. Middle left panel: qRT-PCR analysis revealing change of p24 mRNA levels. Middle right panel and lower panel: representative western blot and densitometry analysis revealing change of p24 protein levels. **(C)** Jurkat cells were transfected with indicated RNAi. 24 hours post-transfection, cells were infected with HIV pseudovirus. 24 hours post-infection, cells were harvested to isolate total RNA and protein, and culture supernatants were collected. Upper left panel: qRT-PCR analysis showing different dose of HSP14 knockdown in Jurkat cells. Upper right panel: Culture supernatants were examined for amount of virus produced using p24 antigen capture ELISA. Middle left panel: qRT-PCR analysis revealing change of p24 mRNA levels. Middle right panel and lower panel: representative western blot and densitometry analysis revealing change of p24 protein levels. **(D)** CEM cells were transfected with indicated RNAi as Jurkat cells. Upper left panel: qRT-PCR analysis showing different dose of HSP14 knockdown in CEM cells. Upper right panel: Culture supernatants were examined for amount of virus produced using p24 antigen capture ELISA. Middle left panel: qRT-PCR analysis revealing change of p24 mRNA levels. Middle right panel and lower panel: representative western blot and densitometry analysis revealing change of p24 protein levels. Control: cell lines were transfected with vector plasmids as control. Blank: cell lines were not transfected with any plasmids as blank control. +: 500ng/μl of HSPA14 overexpression plasmid, ++: 1μg/μl of HSPA14 overexpression plasmid, +++: 2μg/μl of HSPA14 overexpression plasmid. -: 50nM of HSPA14 shRNA plasmi, –: 100nM of HSPA14 shRNA plasmid, —: 200nM of HSPA14 shRNA plasmid.The error bars are presented as the mean ± SD values and significance is defined as *P ≤ 0.05, **P ≤ 0.01 and ***P ≤ 0.001.

According to the evidence we observed in **Figure 2**, we speculate that HSPA14 can also interfere the HIV infection. In order to prove this, we assessed the role of HSPA14 during HIV infection. We examined the effect of HSPA14 on virus production by measuring the expression of viral capsid protein p24. Jurkat cells and CEM cells were transfected with different dose of HSPA14 plasmid or RNAi, followed by HIV pseudovirus infection and different levels assessment of p24. Significant inhibition in a dose-dependent manner of virus production was observed with HSPA14 over-expression in different cells (**Figure 3A, B**). Moreover, HSPA14 knockdown resulted in increased HIV production in a dose-dependent manner in different cells (**Figure 3C, D**). These results suggesting that HSP14 suppress viral gene-expression probably both at transcription level and protein level.

### 3.4 Comparison of HSPAs between acute HIV infected patients with different HIV RNA

**Figure 4:**
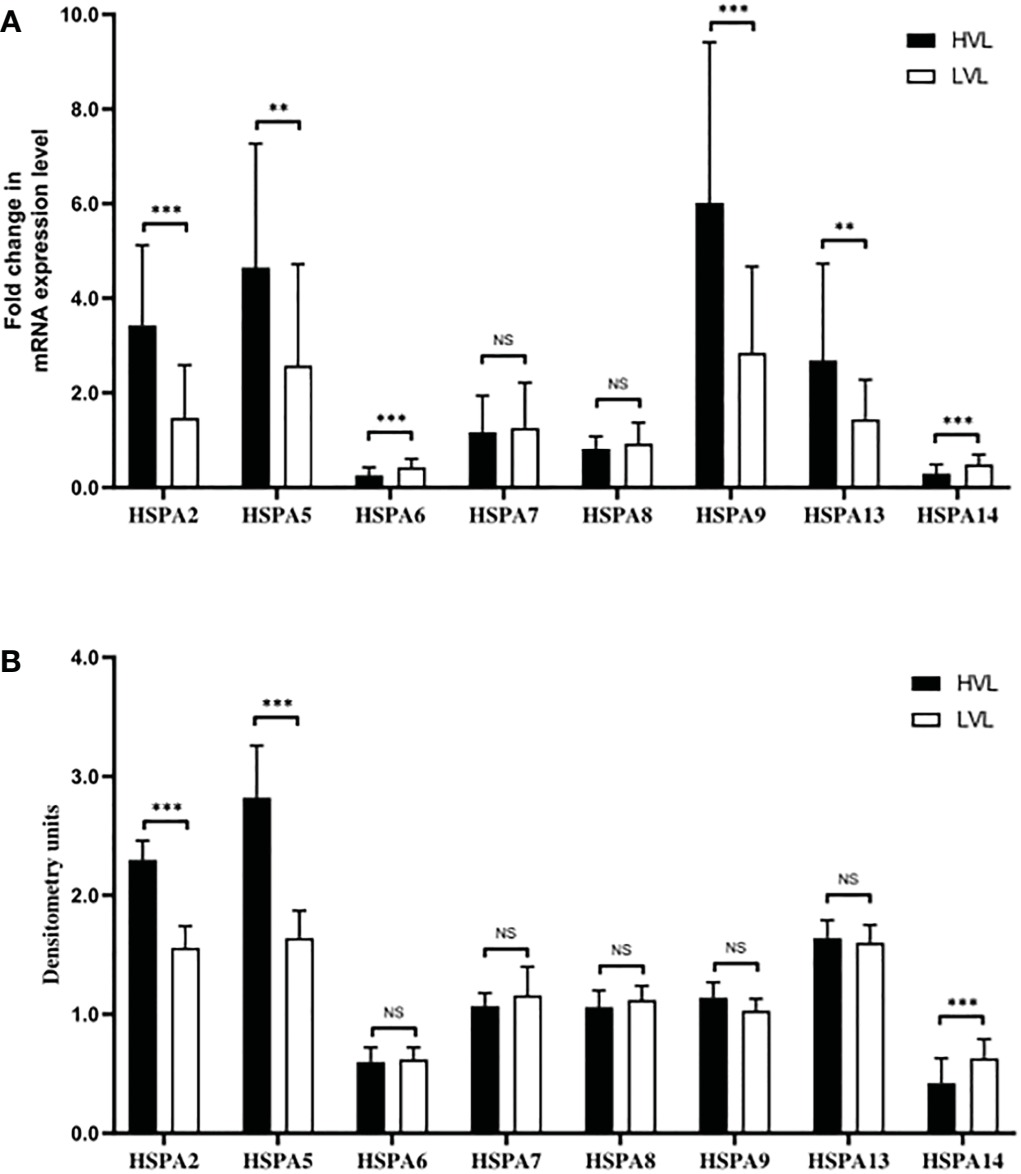
Different expression levels of HSPAs in HVL and LVL patients. CD4+ T cells from PBMC of 70 patients were isolated and harvested for RNA and protein lysate preparation. **(A)** Analyzing and comparing the different HSPA isoforms (including HSPA2, HSPA5, HSPA6, HSPA7, HSPA8, HSPA9, HSPA13 and HSPA14) transcription levels between the LVL and HVL patients by qRT-PCR. **(B)** Comparing the different HSPA isoforms protein levels between the LVL and HVL patients by western blot and densitometry analysis. Significance is defined as NS: P>0.05, *P ≤ 0.05, **P ≤ 0.01 and ***P ≤ 0.001.

We have observed that the HIV infection can suppress the HSPA14 and HSPA14 can interfere the HIV replication *in vitro*. To further confirm the above findings *in vivo*, we examined differences in the expression levels of eight HSPAs, including HSPA14, in peripheral blood CD4+ T cells from untreated acute HIV infected patients with similar general statue but different HIV RNA levels. In HVL patients, HSPA2 and HSPA5 were found to have higher transcript and protein expression levels, while HSPA9 and HSPA13 only had higher transcript levels. HSPA14 had lower transcript and protein expression levels, while HSPA6 and HSPA13 only had lower transcript levels (**Figure 4**). These results are similar to the findings of *in vitro* studies, which further demonstrates the important inhibitory role of HSPA14 in HIV replication. In the same time, we also identify other HSPA isoforms, such as HSPA2 and HSPA5, that may be involved in and affect HIV replication.

## 4 Discussion

In the past few decades, several studies have shown that HSPs play important roles in various viral infections. For example, latent infection of herpes simplex virus-2 (HSV-2) increased the HSPs in human neuroblastoma cells, HSP70 mRNA levels increased in mouse L cells after HSV-1 and HSV-2 infection, and the expression level of HSP70 and HSP90 were increased in human B lymphocytes infected with EB virus (13–15). In addition, HSP70 is associated with the capsid precursor P1 of poliovirus type 1 and coxsackievirus B1 (16), the expression levels of HSP70 and HSP40 in lymphocytes of HIV-1 infected patients increased as well (17). A systematic review has summarized the identified “candidate inhibitors” of various HIV-1 infections, and a variety of subtypes of HSP40s, HSP70s and HSP90s are involved in different stages of the HIV-1 life cycle (18). HSP70 contain 13 kinds of subtypes, several reports have demonstrated the significance of HSP70 subtypes in HIV-1 life cycle (19). HSPA5 is involved in the folding of viral Env proteins in the ER, while other HSPA subtypes interact differently with viral proteins such as Tat, Gag, and Vpr (20–22). Compared with HIV-1CladeC gp120 in astrocytoma, HIV-1CladeB gp120 can induce the expression of HSPA5 and other ER stress markers, and HIV-1Tat can also induce HSPA5 and other ER stress markers (23). HSPA9 was proven to be a necessary helper protein for Nef secretion in the exocrine system. Kammula et al. proved that there was an interaction between HSPA9 and Nef by yeast two-hybrid screening (24).

Although the role of HSP70s in HIV-1 infection is more important than that of other HSPs, further research is needed to clarify the specific function of each subtype. For example, although HSPA8 has been shown to play an important role in replication and assembly of other viruses, its role in HIV-1 infection remains unclear (25, 26). HSPA2 and HSPA1L do not show clear changes in other viral infections or HIV-1 infections, because they are enriched in testicular tissue, which may play vital roles in the process of HIV-1 invasion (27, 28). In addition, although HSPA13 has been shown to interact with the Env protein, the effect of HSPA12, HSPA13 and HSPA14 to HIV-1 are unclear. Thus, this study aims to investigate the role of HSPA14 in HIV-1 infection. We found that the mRNA and protein levels of HSPA14 in the Jurkat, CEM and TZM-bl cells decreased gradually with increasing HIV-1 infection time. Further verification with CD4+ T cells isolated from healthy human peripheral blood showed that the mRNA and protein levels of HSPA14 in the CD4+ T cells infected with HIV-1 pseudoviral particles also decreased with increasing infection time. The above results indicate that HIV-1 replication can inhibit the expression of intracellular HSPA14 at the transcriptional and translational levels *in vitro* infection experiments.

Furthermore, we investigated the effect of HSPA14 on virus replication. The results showed that the mRNA and protein levels of p24 decreased with increasing HSPA14 overexpression in the Jurkat and CEM cell lines overexpressing HSPA14, while the mRNA and protein levels of p24 increased with decreasing HSPA14 expression in the Jurkat and CEM cells infected with HIV-1 pseudovirions. All the above results showed that HSPA14 could inhibit HIV-1 replication. Based on these, we speculate that HSPA14 may act as a transcriptional regulator to inhibit viral replication by directly interacting with cis-acting element binding site on HIV-1 LTR, and may also indirectly inhibit provirus transcription by suppressing transcriptional regulators which promote HIV replication. In addition, HIV-1 might also be developing counteracting strategies to counteract the transcriptional repressive effect caused by HSPA14, such as some viral proteins may reduce intracellular transcription and translation levels of HSPA14 in order to relieve the viral repression by HSPA14.

HspBP1, as a helper protein of HSP70, inhibits its folding activity by binding to the ATPase domain of HSP70 (29). Previous studies have shown that the expression of HspBP1 IgG in the serum of people infected with HIV-1 increases (30). As an intrinsic inhibitor of HIV-1, previous studies have shown that HspBP1 can inhibit HIV-1 replication by competing to inhibit NF-kappa B on the HIV-1LTR promoter. cells (31). In the present study, Co-IP showed that there was a protein-protein interaction between HSPA14 and HspBP1 in both HIV-1 infected Jurkat cells and CEM cells, indicating that the HSPA14 may also participate in regulation of HIV-1 replication by HspBP1.

We further explored the differences in the expression levels of the HSPA subfamily members between HVL patients and LVL patients. The results showed that the transcriptional levels of HSPA2, HSPA5, HSPA9 and HSPA13 were higher and the protein levels of HSPA2 and HSPA5 were also higher in the HVL group. In the LVL group, the transcriptional levels of HSPA6 and HSPA14 were higher and the protein levels of HSPA14 were higher. These results are similar to the findings of previous studies, indicate that besides the HSPA2, HSPA5 and HSPA14 may play important roles in HIV-1 replication, which in ture affects the level of virus replication *in vivo* (32).

## 5 Conclusion

Firstly, we found that the HSPA subfamily of HSPs has important roles. Then, by comparing the expression levels of different HSPA subtypes in patients with different virological levels, we found that HSPA14 is most likely to affect the replication level of HIV-1. Finally, through the *in vitro* cell experiments, we found that the expression level of HSPA14 was inhibited after HIV-1 infection, HSPA14 also inhibited the replication level of the virus and may play a role similar to that of the viral replication inhibitor HspBP1. There are some limitations in this study. First of all, *in vivo* studies of the effect of HSPA14 on HIV-1 replication are lacking due to the limitations of the study conditions. Secondly, the research on the mechanism of HSPA14 affecting HIV-1 replication is superficial, and the interaction between viral proteins (such as Gag, Pol, Env, Tat, Rev, Nef and Vpr) and HSPA14 has not been explored. At the same time, we only explored the effect of HSPA14 on HIV-1 replication by simulating the infection state with HIV pseudovirus, which may be different from the state of the infection state with real HIV-1 virus.Overall, this study showed that HSPA14 can inhibit HIV-1 replication, which provides a research basis for further exploration of its usage as a target for inhibition of HIV replication.

## Data availability statement

The raw data supporting the conclusions of this article will be made available by the authors, without undue reservation.

## Ethics statement

The studies involving human participants were reviewed and approved by Ethics Committee of Tangdu Hospital (Num. 201911-04). The patients/participants provided their written informed consent to participate in this study. Written informed consent was obtained from the individual(s) for the publication of any potentially identifiable images or data included in this article.

## Author contributions

MB, WK, and YS designed the study and collected the blood samples. MB performed the immunoblotting, PCR, and other experiments *in vitro*. WK and YS provided expert guidance and statistical analysis. MB wrote the manuscript. All authors contributed to the article and approved the submitted version.
